# The combined presence of hypertension and vitamin D deficiency increased the probability of the occurrence of small vessel disease in China

**DOI:** 10.1186/s12883-019-1395-2

**Published:** 2019-07-17

**Authors:** Junzeng Si, Kuibao Li, Peiyan Shan, Junliang Yuan

**Affiliations:** 1 Department of Neurology, Qilu Hospital of Shandong University, Jinan, 250012 China; 2 Department of Neurology, Jinan City People’s Hospital, Jinan, 271199 China; 3 Heart Center and Beijing Key Laboratory of Hypertension, Beijing Chaoyang Hospital, Capital Medical University, Beijing, 100020 China; 4 Department of Neurology, Beijing Chaoyang Hospital, Capital Medical University, 8 Gongti South Road, Chaoyang District, Beijing, 100020 China

**Keywords:** 25-hydroxy vitamin D, Small vessel disease, Ischemic stroke, Hypertension

## Abstract

**Background:**

The exact relationship between 25-hydroxyvitamin D [25(OH) D] levels and small vessel disease (SVD) are not clear in China. The aim of this study was to determine such the association between 25(OH) D and SVD in China.

**Methods:**

We retrospectively enrolled 106 patients with SVD and 115 controls between Jan 2017 and Dec 2017. All the subjects were categorized into three subgroups according to the level of 25 (OH) D: vitamin D deficiency (< 12 ng/ml), insufficiency (12–20 ng/ml) and sufficiency (> 20 ng/ml).

**Results:**

Among 106 SVD patients, 80 (75.5%) were men and the mean age was 61.6 ± 13.2 years. The deficiency of 25(OH) D was observed in 76 (71.7%) of SVD patients and 47 (40.9%) of controls (*P* = 0.001). Compared with controls, patients with SVD were more likely to be male, a stroke history, smokers, with hyperlipidemia, higher systolic and diastolic blood pressure and low-density lipoprotein, and lower of 25(OH)D level (*P* < 0.05). Logistic regression analysis revealed the level of 25 (OH) D as an independent predictor of SVD (*OR* 0.772, 95% *CI* 0.691–0.862, *P* = 0.001). Compared with the sufficient 25 (OH) D group, the *ORs* of SVD in deficient and insufficient 25(OH)D group were 5.609 (95% *CI* 2.006–15.683) and 1.077 (95% *CI*: 0.338–3.428) after adjusting for potential confounders, respectively. In hypertensives with vitamin D deficient and insufficient group compared with sufficient group, the *ORs* of SVD increased to 9.738 (95% *CI* 2.398–39.540) and 1.108 (95% *CI* 0.232–5.280), respectively (*P*_interaction_ = 0.001).

**Conclusion:**

We found significant associations between SVD and 25(OH)D deficiency. The combined presence of hypertension and vitamin D deficiency increased the probability of developing SVD. Our findings will warrant further prospective studies in the future.

## Background

Stroke is now recognized to be the second leading cause of death worldwide [1, 2] and the first one in China [3]. Small vessel disease (SVD) is considered to cause 20 to 25% of strokes. According to the STandards for ReportIng Vascular changes on nEuroimaging (STRIVE), there are several neuroimaging features of SVD, including acute small subcortical infarcts, white matter hyperintensities (WMHs), lacunes, microbleeds (CMBs), perivascular spaces (PVS) and brain atrophy [4]. To date, SVD has been recognized to be the second commonest cause of dementia, gait disturbances, affective disorders and late-onset depression [5]. SVD is becoming a major public health issue in Asian countries especially in China [6].

However, SVD is not silent, permanent or untreatable. Identification of controllable and treatable risk factors is essential for effective prevention of SVD. Importantly, accumulating evidence opened new insights and offered new therapeutic targets in recent years. As a fat-soluble vitamin, an increasing body of evidence supported a vital role for vitamin D in brain function and development. Vitamin D may also prevent vascular injury by inhibiting the renin-angiotensin-aldosterone system and atherogenesis [7] and lowering blood pressure. The prevalence of 25-hydroxyvitamin D [25(OH) D] deficiency is high in patients with acute stroke, and it may be associated with greater clinical severity and poor functional prognosis. However, there are only quite a few reports about the exact relationship between 25(OH) D and the occurrence of SVD. It was reported 25(OH)D was inversely associated with lacunes, WMHs and deep CMBs in Korea, which was linked to chronic brain injury associated with SVD [8]. The findings from India also indicated the combined presence of hypertension and vitamin D deficiency increased the probability of developing vascular dementia (VaD) due to SVD, and the intervention of vitamin D status and hypertension could be helpful to reduce the risk of VaD [9]. However, whether these findings could be reproduced in other ethnicities should be further confirmed.

To date, as far as we know, only one study from China reported an association between vitamin D deficiency and total MRI burden of SVD in the south of China [10]. Whether the effect of 25(OH)D on SVD could be modified by the history of hypertension needs to be further elucidated in China. We hypothesized there might be some relationships between 25(OH)D deficiency and the occurrence of SVD in the north of China. Thus, in our present study, we aimed to investigate the exact relationship between 25(OH) D levels and the predictors of SVD in the north of China.

## Materials and methods

### Subjects

Our study was a case-control study, and we retrospectively recruited 106 patients with SVD within 7 days of onset with local neurological deficits lasting more than 24 h and caused by cerebrovascular etiology in the Department of Neurology, Beijing Chaoyang Hospital and Jinan City people’s hospital between Jan 2017 and Dec 2017. The 115 healthy volunteers who were matched with the patients were also recruited. All the subjects were enrolled by randomization and blindness in order to avoid bias.

As for the sample size, vitamin D deficiency (78%) was very common in ischemic stroke in China [11], and the incidence of vitamin D deficiency in the control group was about 60%. We used the following values to calculate the sample size in our study: *α* = 0.05, *β* = 0.20, p1_=_78% (the proportion of vitamin D deficiency in SVD), p0_=_60% (the proportion of vitamin D deficiency in controls), q0 = 1-p0 = 40%, q1 = 1-p1 = 22%, 1:1 design, two sides, the sample size N was about 100. The following formulae were used, N = 2(*Z*_*α*/2_ + *Z*_*β*_ )^2^(p0q0 + p1q1) /(*p*0 − *p*1)^2^.

Detailed neurological examinations were performed in all subjects. The NIH Stroke Scale (NIHSS) and the Rankin scale were to evaluate the stroke severity and disability after a stroke. Magnetic resonance imaging (MRI) was performed in all the patients to determine the presence of SVD, including lacunar infarction, WMHs, CMBs, and PVS according to STRIVE [4]. The exclusion criteria were: (1) age < 18 years old; (2) unable to perform MRI; (3) cardioembolic source of stroke and more than 50% stenosis of the affected large artery; (4) pre-stroke diagnosis of active or chronic inflammatory diseases, depression or other psychiatric disorders, malignant tumor, intracerebral hemorrhage, thyroid diseases, autoimmune diseases, and a history of any central nervous system disease. Our work was approved by the Ethics Committee of Beijing Chaoyang Hospital and Jinan City people’s hospital. Written informed consent was obtained from all participants.

### Clinical variables

We obtained the following clinical data: age, gender; vascular risk factors such as hypertension, diabetes mellitus, hyperlipidemia, stroke, coronary heart disease, hyperlipidemia, atrial fibrillation, peripheral arterial disease and smoking. The laboratory blood tests were obtained including the counts of red blood cell, white blood cell, platelet, fibrinogen, fasting blood glucose, hemoglobin A1c, glycated albumin, uric acid, homocysteine, total cholesterol, low-density lipoprotein, high-density lipoprotein, triglyceride, C-reactive protein, albumin, 25-hydroxy vitamin D. As for the vitamin D level, serum levels of 25(OH)D were measured using enzyme immunoassay kits. The subjects were stratified into three subgroups: vitamin D deficiency (< 12 ng/ml), insufficiency (12–20 ng/ml) and sufficiency (> 20 ng/ml) [9, 12], according to the guidelines of the National Osteoporosis Society [12].

### Statistical analysis

The data were described using the mean and standard deviation values for continuous variables, the median and interquartile range values for categorical variables, and absolute numbers and percentages for nominal and categorical variables, and we compared the groups using the nonparametric Mann-Whitney U test. We performed a chi-square test to determine the correlation between categorical variables and a t-test between continuous variables. The association between 25(OH)D and SVD was tested using logistic regression analyses, and the odds ratios (*ORs*) and 95% confidence interval (*CI*) were estimated. We used the Statistical Package for Social Sciences (SPSS) version 16.0 (SPSS Inc., Chicago, IL, USA) for data analysis. A *P* value less than 0.05 was considered statistically significant.

## Results

A total of 221 subjects were enrolled, and 106 were patients with SVD and 115 for controls. Among 106 SVD patients, 80 (75.5%) were men and the mean age was 61.6 ± 13.2 years. The scores of NIHSS and Rankin scale in SVD group were 3 (1–5) and 1 (0–2), respectively. The demographics, vascular risk factors, and the laboratory findings were presented in Table 1. Compared with controls, patients with SVD were more likely to be male, smokers, with hyperlipidemia, higher systolic and diastolic blood pressure, higher low-density lipoprotein, and a stroke history (*P* < 0.05).

**Table 1:** The baseline demographics, clinical characteristics and biochemical variables between the two groups

Variables	SVD (*N* = 106)	Control (*N* = 115)	*P*
*Demographics*			
Age (Y)	61.6 ± 13.2	60.5 ± 11.7	0.516
Sex (Male, %)	80(75.5%)	54(47%)	0.001*
Systolic blood pressure	151.5 ± 21	133.7 ± 17.3	0.001*
Diastolic blood pressure	87 ± 14.2	80.1 ± 12.3	0.001*
*Risk factors*			
Hypertension	66(62.3%)	75(65.2%)	0.648
Diabetes mellitus	33(31.1%)	26(22.6%)	0.152
Coronary disease	11(10.4%)	16(13.9%)	0.423
Prior stroke	26(24.5%)	5(4.3%)	0.001*
Hyperlipidaemia	79(74.5)	31(27%)	0.001*
Atrial fibrillation	2(2.3%)	2(6.7%)	0.251
Peripheral arterial disease	6(5.7%)	3(2.6%)	0.252
Smoking	60(56.6%)	45(39.1%)	0.010*
*Blood tests*			
White blood cell (10^9^/L)	7.1 ± 2.1	6.9 ± 2.1	0.514
Red blood cell (10^12^/L)	4.6 ± 0.6	4.6 ± 0.6	0.773
Platelet (10^9^/L)	210.8 ± 92.6	221.7 ± 55.7	0.290
Total cholesterol (mmol/L)	4.4 ± 0.9	4.3 ± 0.8	0.259
Low-density lipoprotein (mmol/L)	2.7 ± 0.8	2.4 ± 0.8	0.003*
High-density lipoprotein (mmol/L)	1.1 ± 0.3	1.1 ± 0.3	0.103
Uric acid(μmol/L)	329.8 ± 87.9	330.3 ± 95.9	0.972
Homocysteine(μmol/L)	18.9 ± 10.5	18 ± 12.3	0.610
Fasting blood glucose (mmol/L)	5.9 ± 1.8	5.6 ± 1.3	0.231
Hemoglobin A1c (%)	6.4 ± 1.4	6.3 ± 1.2	0.737
Glycated albumin(%)	13.8 ± 2.4	17.5 ± 3.8	0.060
Fibrinogen(mg/dl)	268.5 ± 82.2	271.8 ± 87.3	0.843
C-reactive protein (mg/L)	2.05(1.2~4.0)	2.25(0.89~4.56)	0.925
25-hydroxy vitamin D (ng/mL)	10.2 ± 5.6	14.6 ± 7.5	0.001*
Albumin (g/L)	42.1 ± 4.4	42.5 ± 4.8	0.681

Values represent number (percent) for categorical variables and mean ± SD or median (interquartile range) for continuous variables. *P < 0.05 is statistically significant

The levels of 25 (OH) D were classified as sufficient in 11 patients (10.4%) vs 25 controls (21.7%), insufficient in 19 patients (17.9%%) vs 43 controls (37.4%), and deficient in 76 patients (71.7%) vs 47 controls (40.9%). The mean serum 25(OH)D level was much lower in SVD patients (10.2 ± 5.6 ng/ml) compared with controls (14.6 ± 7.5 ng/ml) (*P* = 0.001).

Logistic regression analysis revealed the level of 25(OH)D as an independent predictor of SVD (*OR* 0.772, 95% *CI* 0.691–0.862, *P* = 0.001) after adjusting for the other covariates, shown in Table 2. Compared with the sufficient group of 25(OH)D, we also found the *ORs* of SVD in the deficient and insufficient group were 5.609 (95% *CI* 2.006–15.683, *P* = 0.001) and 1.077 (95% *CI* 0.338–3.428, *P* = 0.9) after adjusting for potential confounders, respectively (Table 3).

**Table 2:** Multivariate logistic regression as predictors of the occurrence of SVD

Predictors	*P*	*OR*	95% *CI* for *OR*
Sex	0.127	0.245	0.040–1.493
Hyperlipidemia	0.862	0.875	0.194–3.947
Prior Stroke	0.070	5.231	0.874–31.310
Smoking	0.061	0.131	0.016–1.095
Systolic blood pressure	0.696	1.008	0.967–1.052
Diastolic blood pressure	0.750	0.990	0.928–1.056
Low-density lipoprotein	0.170	0.585	0.272–1.258
25-hydroxy vitamin D	0.001*	0.772	0.691–0.862

*P < 0.05 is statistically significant

**Table 3:** Association of vitamin D with SVD estimated by odds ratio (*OR*) at 95% confidence interval (*CI*)

Vit D (ng/ml)	SVD(106)	Control(115)	Model 1	Model 2
≥20	11(10.4%)	25(21.7%)	1(Ref)	1(Ref)
12–20	19(17.9%)	43(37.4%)	0.740 [1.171(0.461–2.972)]	0.9 [1.077(0.338–3.428)]
≤12	76(71.7%)	47(40.9%)	0.001* [4.154 (1.797–9.599)]	0.001*[5.609(2.006–15.683)]

Model 1: adjusted with age and gender;

Model 2: additionally adjusted with hyperlipidemia, stroke, smoking history, systolic blood pressure, diastolic blood pressure, low-density lipoprotein, diabetes, hypertension

*P < 0.05 is statistically significant

We also found there was a significant interaction between vitamin D status and hypertension on the presence of SVD (*P*_interaction_ = 0.001). Compared with sufficient group, in hypertensives with vitamin D deficient and insufficient group, the *ORs* increased to 9.738 (95% *CI* 2.398–39.540, *P* = 0.001) and 1.108 (95% *CI* 0.232–5.280, *P* = 0.898), respectively (Table 4). However, we did not find such interaction between vitamin D status and hypertension in non-hypertensive patients.

**Table 4:** Odds ratio (*OR*) at 95% confidence interval (*CI*) of SVD in hypertensives for three groups of serum 25(OH)D

Vit D (ng/ml)	Model 1		Model 2		*P*-interaction
	*P*	*OR* (95% CI)	*P*	*OR* (95% CI)	
Hypertensive					0.001*
≥ 20	1(Ref)		1(Ref)		
12–20	0.980	0.985(0.294–3.295)	0.898	1.108(0.232–5.280)	
< 12	0.002*	5.461(1.0–15.651)	0.001*	9.738(2.398–39.540)	

Model 1: adjusted with age and gender;

Model 2: additionally adjusted with hyperlipidemia, stroke, smoking history, systolic blood pressure, diastolic blood pressure, low-density lipoprotein, diabetes, hypertension

*P < 0.05 is statistically significant

## Discussion

In our present study, we found significant associations between SVD and 25(OH)D deficiency. We also found there was a significant interaction between vitamin D status and hypertension in patients with SVD. Random clinical trials should also focus on supplementation of vitamin D in subjects with vitamin D deficiency and particularly those with a history of hypertension.

As a neurosteroid, vitamin D plays a vital role in preventing vascular injury through the mechanism of inhibiting the renin-angiotensin-aldosterone system and atherogenesis [7], lowering blood pressure, reduction in endothelial dysfunction [13], and neuroprotective actions in stroke [14]. Accumulating data have supported the hypothesis lower 25(OH)D level was associated with ischemic stroke, higher mortality and poor outcome [15–26], and a higher vitamin D was linked to a decreased risk of cerebrovascular disease [27]. These findings were also substantiated by the evidence of meta-analysis [28]. However, there are very limited studies linking SVD and vitamin D [29, 30]. Recently, some cross-sectional studies proved that 25(OH)D was inversely associated with lacunes, WMHs and deep CMBs [8]. The findings from India also revealed that deficient vitamin D was associated with a 2.2-fold increase in odds of small-vessel related dementia [9]. As for the total burden of SVD, lower 25(OH)D was associated with greater total SVD burden in ischemic stroke [10]. However, the above studies had a limited representation of individuals from China. In our study, we further confirmed patients with SVD were correlated with the deficiency of 25(OH)D. Our study was in line with the former studies from different races including in Nepal, India, Iran and Italy [15, 31–37].

SVD is associated with vascular risk factors, among which hypertension is considered as an important, preventable risk factor for cardiovascular disease and stroke. Several physiological mechanisms link vitamin D to hypertension because calcitriol acts as an endogenous inhibitor of the Renin-Angiotensin System. There is a growing body of evidence about the association between vitamin D and hypertension in different ethnicities. In Indian population, an inverse association was found between 25(OH)D and risk of ischemic stroke, which indicated the management of hypertension and severe vitamin D deficiency, particularly in hypertensive subjects, could be helpful to prevent stroke effectively [38]. Another observational study also supported a close relationship between 25(OH)D and higher blood pressure [39]. Our results are consistent with the prior studies [9, 38], with the evidence that hypertension may amply aggravate the risk of SVD with lower vitamin D levels [40]. Our findings were also in accordance with the evidence of cardiovascular disease from our research team (Li et al) [41]. As known, both hypertension and vitamin D deficiency are controllable and treatable parameters, thus, monitoring and management of vitamin D and hypertension may reduce the risk of SVD.

However, the mechanism of deficiency of vitamin D and the developing of SVD is not fully understood. It has been reported vitamin D may play an important role in neuroprotection, perhaps through detoxification pathways, stimulation of neurotrophic factors, inhibition of inducible nitric oxide synthase, antioxidation/anti-inflammatory, neuronal calcium regulation, and enhanced nerve conduction [42]. Serum vitamin D was inversely associated with the levels of interleukin-6 and C reactive protein, suggesting a potential anti-inflammatory role for vitamin D underlying in stroke [43, 44]. It is also plausible that vitamin D supplementation could be a beneficial intervention for stroke prevention. Both hypertension and SVD may also have a close relationship with arterial stiffness [45], which may be also due to the deficiency of vitamin D [46], however, data from randomized clinical trials are needed to clarify these speculations [47].

Our study also had some limitations. First, this study was designed with limited samples especially after categorized into three subgroups, and we could not prove a causal relationship between 25(OH)D and SVD. Studies with larger numbers from multiple centers in China are needed urgently to further confirm our findings. Second, the multiple other factors such as nutrition status, physical activity, serum calcium and phosphate, health education, social status, inflammatory markers, seasonal categories were not available in our study [9, 38, 48]. In addition, serum parathyroid hormone and alkaline phosphatase must be taken into account when considering vitamin D metabolism [49, 50]. Furthermore, vitamin D receptor gene polymorphism may protect against neurological deficits and neuronal death, however, we did not test vitamin D receptor activation in our study [51]. Third, we did not classify SVD into different types according to STRIVE such as lacunes, WMHs, CMBs or PVS [8, 52], and we did not assess the total SVD burden (0 to 4) [10]. Further studies are required to confirm this association and explore the association among different subtypes of SVD [8, 17]. Fourth, there were some different categories according to levels of vitamin D (stratified into 2 or 3 or 5) in different studies [38, 53], such as deficiency (< 25 nmol/L or < 20 ng/mL), insufficiency (25–50 nmol/L or 20–30 ng/mL), sufficiency (≥50 nmol/L or ≥ 30 ng/mL) [44, 54, 55]. In spite of these limitations, to the best of our knowledge, this is the first study to report a link between vitamin D status and hypertension associated with SVD in the north of China. However, our findings also need to be validated in a larger study in other regions in China.

## Conclusion

We found significant associations between SVD and 25(OH)D deficiency. The combined presence of hypertension and vitamin D deficiency increased the probability of SVD. Our findings will warrant further prospective studies or interventional studies with large samples in the future.

## Data Availability

The datasets used and/or analyzed during the current study are available from the corresponding author upon request.

## Abbreviations

25(OH) D: 25-hydroxyvitamin D
CI: confidence interval
CMBs: microbleeds
OR: odds ratio
PVS: perivascular spaces
STRIVE: the STandards for ReportIng Vascular changes on nEuroimaging
SVD: small vessel disease
VaD: vascular dementia
WMHs: white matter hyperintensities
